# Failure of Long-Term Memory Formation in Juvenile Snails Is Determined by Acetylation Status of Histone H3 and Can Be Improved by NaB Treatment

**DOI:** 10.1371/journal.pone.0041828

**Published:** 2012-07-25

**Authors:** Alexandra B. Danilova, Larisa N. Grinkevich

**Affiliations:** Laboratory of regulation of functions of brain neurons, Pavlov Institute of Physiology Russian Academy of Sciences, St. Petersburg, Russia; Tokai University, Japan

## Abstract

**Background:**

Animals’ capacities for different forms of learning do not mature simultaneously during ontogenesis but the molecular mechanisms behind the delayed development of specific types of memory are not fully understood. Mollusks are considered to be among the best models to study memory formation at the molecular level. Chromatin remodeling in developmental processes, as well as in long-term memory formation, was recently shown to play a major role. Histone acetylation is a key process in the chromatin remodeling and is regulated through the signaling cascades, for example MAPK/ERK. Previously, we found that MAPK/ERK is a key pathway in the formation of the food aversion reflex in Helix. Pretreatment with upstream ERK kinase inhibitor PD98059 prevented food avoidance learning in adult Helix. In contrast to adult snails, juveniles possess immature plasticity mechanisms of the avoidance reflex until the age of 2–3 months while the MAPK/ERK cascade is not activated after aversive learning. In the present study, we focused on the potential MAPK/ERK target - histone H3.

**Methodology/Principal Findings:**

Here we found that a significant increase in histone H3 acetylation occurs in adult animals after learning, whereas no corresponding increase was observed in juveniles. The acetylation of histone H3 is regulated by ERK kinase, since the upstream ERK kinase inhibitor PD98059 prevented the increase of histone H3 acetylation upon learning. We found that the injection of histone deacetylase inhibitor sodium butyrate (NaB) prior to training led to induction in histone H3 acetylation and significantly ameliorated long-term memory formation in juvenile snails.

**Conclusions/Significance:**

Thus, MAPK/ERK-dependent histone H3 acetylation plays an essential role in the formation of food aversion in Helix. Dysfunction of the MAPK/ERK dependent histone H3 acetylation might determine the deficiency of avoidance behavior and long-term plasticity in juvenile animals. Stimulation of histone H3 acetylation in juvenile animals by NaB promoted avoidance plasticity.

## Introduction

Despite extensive research on molecular mechanisms of formation, consolidation and retention of memory traces in the brain, the major phenomena in neurobiology, i.e. learning and memory, are poorly understood. It has been shown that long-term memory formation is determined by a rearrangement of neuronal networks and by an increase in efficiency of synaptic contacts between neurons. These processes require modulation of the genome [1], [2], [3], [4], [5]. Despite the fact that neurons are non-proliferating cells, protein kinase cascades and proteins involved in cellular proliferation and differentiation (ERK, p38, JNK, CREB, c-fos, zif268) are important for long-term memory formation [5], [6], [7], [8], [9]. Moreover, it was recently shown that epigenetic remodeling is also involved in learning and memory, in the same way as in cellular differentiation and development [5].

It is well-known that animals capacities for different forms of learning do not mature simultaneously during ontogenesis. Long-term memory of aversive behavior is the last to develop during ontogenesis [10], [11], [12], [13]. The molecular mechanisms responsible for the delayed development of specific types of learning are not fully understood and comparative ontogenetic approaches allow dissecting key players in plasticity mechanisms for some forms of learning.

Remodeling of the chromatin during development and long-term memory formation has been become a focus of research only recently. Histone modifications, such as acetylation and phosphorylation, as well as DNA methylation, were shown to play an essential role in chromatin remodeling and are critically important for transcriptional regulation during memory consolidation [6], [7], [8], [9]. Acetylation of histone H3 at Lys14 is a key process in chromatin remodeling and consequently in induction of transcription [6], [7], [8]. Histone acetylation relaxes chromatin, opening the promoter regions for transcription factors, thus recruiting RNA synthesis complex. At the same time phosphorylation and acetylation of the histones are regulated through intracellular signal cascades, such as MAPK/ERK (mitogen-activated protein kinase/extracellular signal-regulated kinase) [6], [7], [8], [9]. The level of histone acetylation can be tuned by histone acetyltransferases (HAT) and histone deacetylases (HDAC). It is suggested that the MAPK/ERK-dependent acetylation of histones can be mediated by the CREB-binding protein (CBP), a well-known transcription activator, which possesses endogenous HAT activity [14], [15]. Mutations in the CBP encoding gene result in the Rubinstein-Taybi syndrome in humans, which is characterized by mental retardation [15]. Importantly, long-term memory can be influenced by the induction of the histone acetylation by HDAC inhibitors [6], [7], [9], [14], [16].

Mollusks are a commonly used model for the research of learning and memory due to the relative simplicity of their CNS and stereotyped behavior. Since serotonin plays an important role in plasticity of the avoidance behavior in mollusks [3], [12], [17], it is suggested that formation of long-term sensitization and conditioned avoidance reflexes in mollusks are associated with the course of development of the serotoninergic system [12], [18], [19], [20]. We hypothesized that immaturity of the serotonin signal transduction underlies the inability of juvenile mollusks *Helix* to form long-term types of avoidance behavior [13]. Previously, we showed that the MAPK/ERK regulatory cascade mediates serotonin signaling and plays the key role in the formation of the food aversion reflex in *Helix* [13], [20], [21], [22]. Recently we found that upon learning, in adult *Helix,* there is a MAPK/ERK dependent asymmetric increase of histone H3 acetylation in the command neurons of the right parietal ganglia RPa(2/3), but not in the symmetrical command neurons of the left parietal ganglia LPa(2/3) [23]. Parietal ganglia together with visceral, pleural and pedal ganglia compose subesophaegal complex of ganglia, the major part of *Helix* CNS.

Juvenile animals, in contrast to adults, possess immature mechanisms of long-term plasticity of avoidance reflexes and showed a significantly lower degree of MAPK/ERK phosphorylation and the absence of its activation after training in the subesophageal complex of ganglia, which is the major part of *Helix* CNS [13]. Moreover, the juvenile snails differ from the adults in the spectrum of MAPK/ERK induced transcription factors (TFs) binding DNA regulatory elements SRE and AP-1 [20].

Taking into account that MAPK/ERK-dependent regulation of gene expression is mediated not only through the activation of the specific TFs [3], [24], but also through posttranslational modifications of histones [6], [7], [8], [9], we suggested that in juvenile animals MAPK dysfunction may result not only in the TFs disturbance, but also in the deficiency of histones modifications.

To investigate the involvement of histones modifications in the long-term memory formation in *Helix* ontogenesis we carried out a comparative study of histone H3 acetylation in the adult and juvenile snails CNS (subesophageal complex of ganglia) after food aversion learning. To our knowledge, such studies have not been performed before.

We demonstrated a significant MAPK-dependent increase of H3 histone acetylation in the subesophageal complex of ganglia of adult animals after learning, whereas in juvenile snails under the same conditions histone H3 acetylation induction was not observed. Moreover, we found that injection of histone deacetylase inhibitor sodium butyrate (NaB) prior to training elevated the level of histone acetylation and significantly impoves the long-term memory formation in the juvenile snails. Thus, histone H3 acetylation plays an essential role in food aversion formation in *Helix*. Dysfunction of histone H3 acetylation through a lack of the expression of certain genes might determine the deficiency of avoidance behavior and long-term plasticity in juvenile animals.

## Results

### Histone H3 Acetylation in the Subesophageal Complex of Ganglia of Adult Helix after Food Aversion Learning

**Figure 1:**
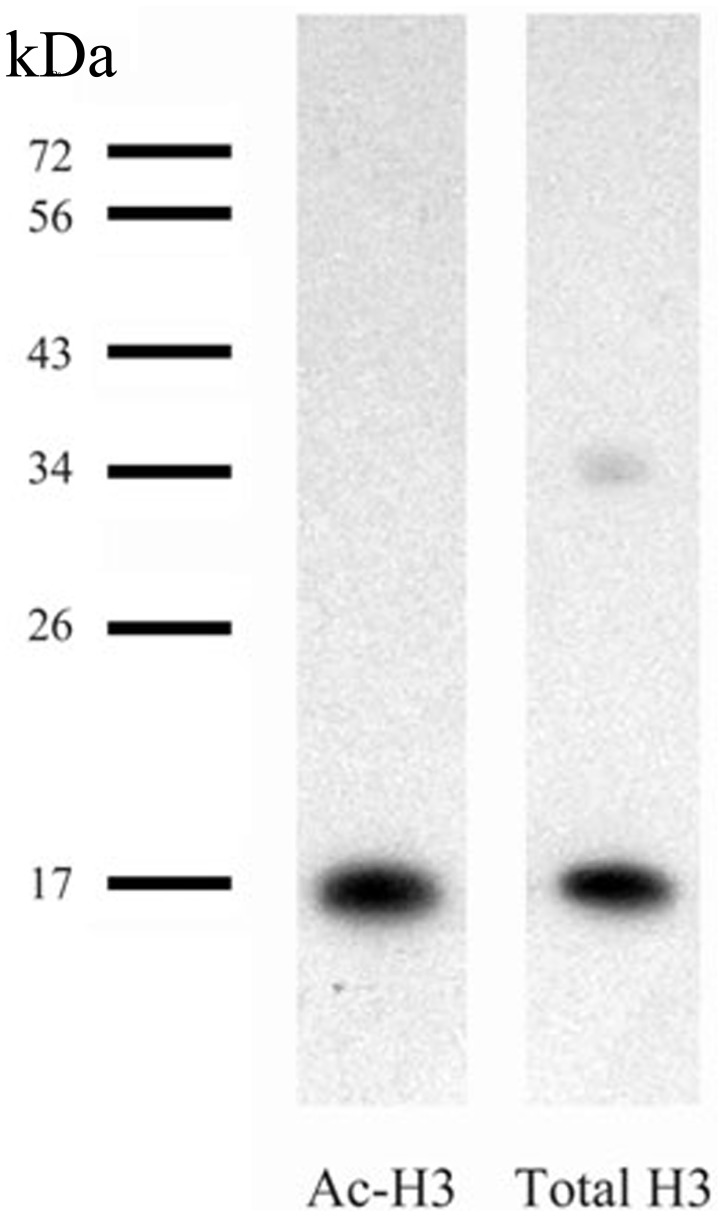
Western blots of lysates from Helix subesophageal complex of ganglia. Total H3 histone and acetyl-H3 histone antibodies showed high specificity to a single component in extracts of *Helix lucorum* CNS.

Histone H3 is known to be a highly conserved protein present both in vertebrates and invertebrates [9], [25]. Using the antibody against the total histone H3, we detected by Western blot a single band of 17 kDa in subesophageal complex of ganglia of *Helix* CNS (Fig. 1), which corresponds to histone H3 of different animal species [25]. Antibodies against acetyl-histone H3 also revealed only one band of protein in *Helix* the subesophageal complex of ganglia of CNS (Fig. 1). All antibodies failed to show any unspecific binding to other proteins in Western blots.

To determine the role of H3 histone in memory formation in *Helix*, we carried out the analysis of acetylation of H3 histone in the subesophageal complex of ganglia of CNS, where neurons involved in control of avoidance behavior are located [17]. Snails from each group were killed 1 h after training, and the ratio between ac-H3 histone and total - H3 histone levels in the subesophageal complex of ganglia was determined using Western blot. Naïve snails were used as a control.

**Figure 2:**
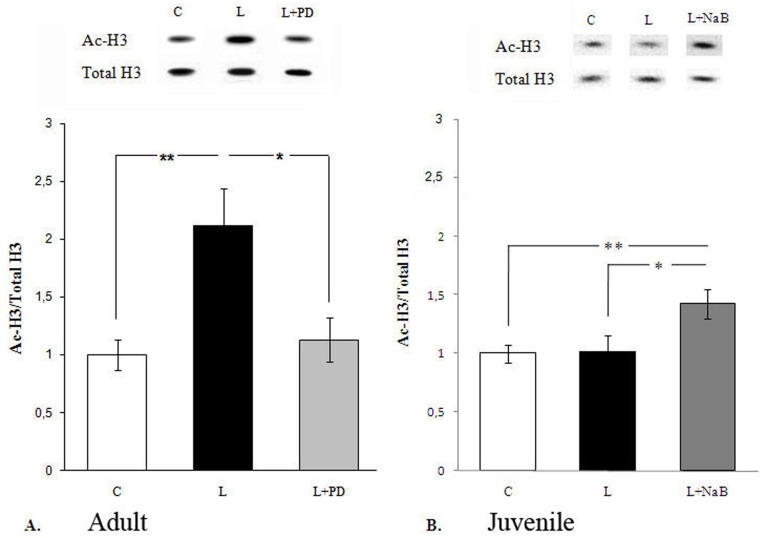
Histone H3 acetylation in the subesophageal complex ganglia of the snails after food aversion training. A. Increasing of histone H3 acetylation in the subesophageal complex ganglia of the adult snails is associated with activation of ERK-signaling pathway after aversive learning. The amount of acetylated histone H3 was significantly higher after learning (L), compared to control animals (C). The acetylation was blocked with upstream kinase inhibitor PD98059. The ratio of acetylated histone H3 (Ac-H3) to the total H3 (total H3) determined after Western blot analysis was normalized with average ratio of Ac-H3/total H3 of the naive adult samples from the same gel (2–3 samples of the naive adult group were analyzed in each gel). Group data show mean ± SEM normalized ratios of Ac-H3/total H3. **C** - Control animals pretreated with vehicle; **L** - Learning animals pretreated with vehicle; **L+PD** – Learning animals pretreated with the MEK inhibitor PD98059. Number of independent experiments: C (n = 12); L (n = 12); L+PD (n = 7). * - p<0.01; ** - p<0.002. Above each histogram: representative Western blots. B. Histone H3 acetylation in the subesophageal complex ganglia of the juvenile snails after food aversion training and NaB injection. No change in the histone H3 acetylation was observed in the juvenile snails after training (L). The injection of HDAC inhibitor **NaB** increased the histone H3 acetylation in the juvenile snails after training (L+NB). **C** - Animals injected with saline; **L** - Learning animals injected with saline; **L+NB** – Learning animals injected with NaB. Number of independent experiments: C (n = 9); L (n = 7); L+NB (n = 8). *p<0.02; **p<0.002.

We observed a significant increase of histone H3 acetylation in the subesophageal complex of ganglia after learning (Fig. 2A) (F(2,28) = 6.8376, p<0.0038 one-way Anova; post hoc Fisher–test: control versus learning p<0.002), while the total amount of H3 histone did not change.

Taking into account the importance of the MAPK/ERK pathway during food aversion learning of *Helix* [13] and the increase of histone acetylation in CNS upon learning (Fig. 2), we investigated whether ERK regulates histone acetylation during long-term memory formation in *Helix*. We injected animals with an upstream ERK kinase inhibitor PD98059 (40 µM) 30 minutes prior to training. This concentration of the inhibitor abolished learning and prevented ERK activation in *Helix* [13]. As shown in Fig. 2A, a pretreatment with PD98059 prevented induction of histone H3 acetylation (F(2, 28) = 6.8376, p<0.0038 one-way Anova; post hoc Fisher–test: learning versus learning + PD p<0.014).

Thus, there is a significant MAPK/ERK dependent increase of histone H3 acetylation during formation of food aversion learning in the subesophageal complex of ganglia in the CNS of adult *Helix*.

### Histone H3 Acetylation in the Juvenile Snails after Food Aversion Training and Treatment with HDAC Inhibitor NaB

Next, we performed analysis of histone H3 acetylation in the subesophageal complex of ganglia of the juvenile *Helix* after training. Two-three months old juvenile snails, which are unable to acquire defensive types of plasticity (sensitization and avoidance conditioning) [12], were used in the experiments. Since ganglia of juvenile snails are small in size, we combined ganglia from two or three animals for each sample.

We showed that juvenile animals, unlike the adults, demonstrated no increase in histone H3 acetylation in the subesophageal complex of ganglia after learning (Fig. 2B). F(1,14) = 0.02 p = 0.89 one-way Anova.

Considering the findings in vertebrates on possible memory improvement through the induction of acetylation [14], [16] by administration of HDAC inhibitors, we hypothesized that the deficit of long-term memory in juvenile snails might be also alleviated through the administration of HDAC inhibitors. It was shown that HDAC inhibitors increase histone acetylation in animals capable for learning [6], and in animals with cognitive deficit [14], [16]. Therefore, we investigated: 1) whether the administration of HDAC inhibitors sodium butyrate in juvenile snails may lead to the increased histone H3 acetylation in their CNS; 2) whether the long-term (avoidance) memory deficit can be improved in these animals.

One hour before training we injected juvenile snails with HDAC inhibitor sodium butyrate (NaB) 1.2 g/kg and measured histone H3 acetylation in the the subesophageal complex of ganglia in CNS 1 h after training. Based on the work of Fischer and colleagues [16] where it was shown that NaB treatment recovered learning capabilities in animals with heavy neurodegeneration, we decided to choose NaB for our experiments as well. We found that the injection of NaB stimulates histone H3 acetylation in the subesophageal complex of ganglia of trained juvenile snails (Fig. 2B). The increase in H3 acetylation of trained animals injected with NaB is significant in comparison to trained animals injected with saline, F(2,21) = 6.34, p<0.007 one-way Anova; *p<0.02 post hoc Fisher–test: learning versus learning + NaB; **p<0.002. post hoc Fisher–test: control versus learning + NaB.

Thus, treatment of juvenile snails with HDAC inhibitor sodium butyrate leads to an increase of histone H3 acetylation. The increase in H3 acetylation in adult animals and consequent long-term memory formation, prompted us to investigate, whether increase in H3 acetylation by NaB injection will stimulate long-term memory in young snails.

To check our hypothesis, we tested the aversive behavior of juvenile snails that had been pretreated with saline or NaB 1 h prior training. The percentage of aversive responses to conditional stimulus (carrot) was quantified. The reaction was measured 48 h after the training procedure and was considered aversive if the animal avoided the carrot for more than two minutes.

Cross-tabulation tables with 2 sided Fisher’s exact test were used for comparison of probabilities of behavior. In the control group of saline injected animals food refusal was found in 3 out of 18 snails (16.7%). In the group injected with the HDAC inhibitor NaB food refusal was found in 10 out of 18 animals (55.6%). Fisher’s exact test yielded p<0.035. It is noteworthy that in the group of adult snails which were not pretreated with NaB before learning, food refusal was demonstrated in 80%–90% of all cases.

**Figure 3:**
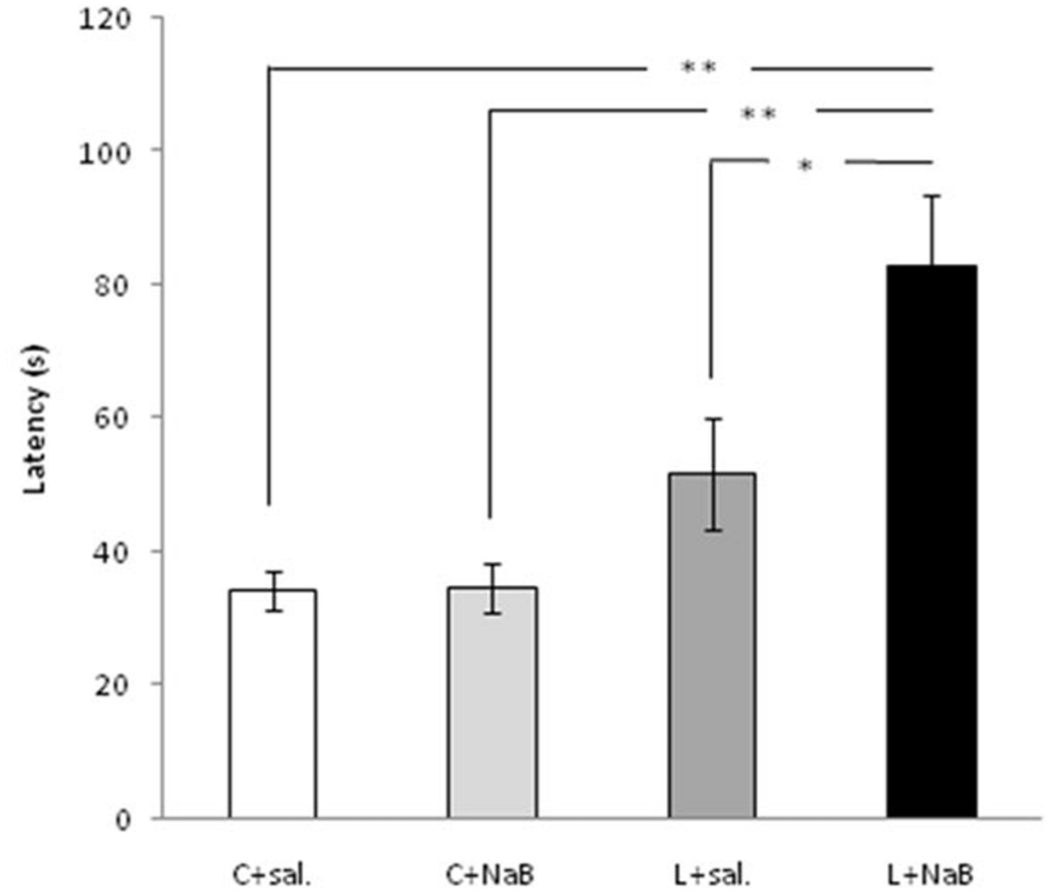
Latency periods of consummatory reaction in the juvenile Helix snails after training. The injection of HDAC inhibitor **NaB** increased the latency period of consummatory reaction in juvenile snails 48 h after training. Latency of animals, avoiding food was estimated equal to 120s. The animals were not tested for longer times. Usually they moved away from the food. **C+sal** - Animals injected with saline (*n* = 18); **C+ NaB** - Animals injected with NaB (*n* = 18); **L+sal** - Learning animals injected with saline (*n* = 18); **L+NaB** – Learning animals injected with NaB; (*n* = 18) *p<0.025; **p<0.0001. Error bars indicate SEM. An asterisk and two asterisks denote significant differences (*p*<0.03 and *p*<0.0001 as followed) as determined by Fischer’s and Tukey’s multiple-comparison tests.

In addition, we estimated a latency of consummatory reactions of the animals (Fig. 3). Latency of the animals, avoiding food for more than 2 minutes, was estimated equal to 120 seconds. The animals were not tested for longer periods. As usual, they moved away from the food and did not come back.

Latency periods of the animals pretreated with saline or NaB prior to learning did not differ (F(1, 34) = 0.00866, p = 0.92642 Anova) (34±3 s and 35±4 s correspondingly) (Fig. 3). Also, no statistically significant differences were found between control animals and trained animals pretreated with saline (C+sal versus L+sal F(1, 34) = 3.8297, p = 0.06). Test was carried out 48 h after training.

Therefore, learning induces significant increases in latency in animals pretreated with NaB in comparison with both control animals treated with NaB (C+NaB versus L+NaB F(1, 34) = 18.876, p<0.00012) and trained animals pretreated with saline (L+sal versus L+NaB F(1, 34) = 5.5010, p<0.02498). Latency of the animals tested 48 h after the procedure was 53±8 s for the snails pretreated with saline and 80±11 s for the snails pretreated with NaB.

Thus, the animals pretreated with NaB showed an increase of aversive reactions 48 h after learning in comparison with the saline pretreated snails. This data demonstrates that HDAC inhibitor sodium butyrate enhances formation of the long-term aversive memory in juvenile animals.

## Discussion

**Figure 4:**
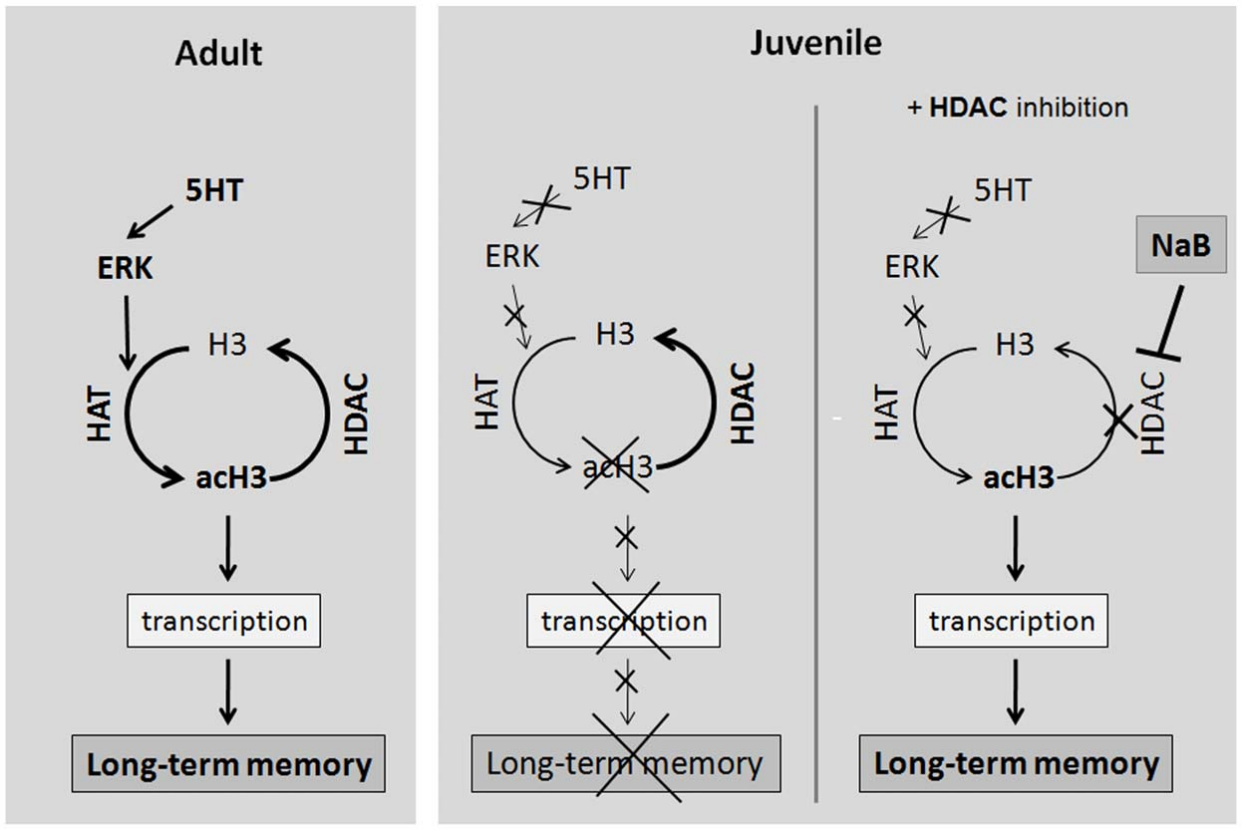
Model. The involvement of epigenetic changes in long-term memory formation in *Helix*. Upon learning there is serotonin (5-HT) - dependent induction of MAPK/ERK pathway and acetylation of downstream target Histone H3 in *Helix*. In juvenile animals, incapable to form long-term aversive behavior upon learning procedure, there is no MAPK/ERK activation and no histone H3 acetylation. These epigenetic changes can be a cause of abnormal transcription crucial for synaptic plasticity and long-term memory formation. Treatment with deacetylase inhibitor NaB induces acetylation and stimulates long-term memory formation in juvenile animals.

Our work shows significant MAPK/ERK-dependent increase of H3 histone acetylation in the subesophageal complex of ganglia of CNS of adult animals after learning, whereas in juvenile snails (possessing immature mechanisms of long-term plasticity of avoidance behavior) under these conditions histone H3 acetylation was not observed. The injection of HDAC inhibitor sodium butyrate prior training induces long-term memory formation in juvenile animals. We summarized our data on the model in Fig. 4. These findings are consistent with a number of studies showing an important role of histone H3 acetylation during long-term memory formation [6], [7], [8]. Induction of histone acetylation has been shown during formation of some types of conditioned reflex both in vertebrates and invertebrates and in the non associative learning model in *Aplysia* [6], [7], [9], [25], [26]. In in vivo models for learning (where it was also used noxious reinforcement) there is an induction of histone H3 acetylation 1 h after conditioning [17]. Our observation that histone acetylation is increased during long-term memory formation in *Helix* also suggests a high level of evolutional conservatism of this phenomenon.

The subesophageal complex of ganglia (2 parietal, 1 visceral, 2 pleural and 1 pedal ganglia) of *Helix* CNS plays an exceptionally important role in defensive behavior of mollusks [6], [12]. Neurons involved in control of avoidance behavior are located in this complex, including sensory cells, motor neurons, modulatory neurons and 9 giant (100–250 µm) premotor withdrawal interneurons (command neurons). All these cells are activated upon noxious stimulation. Modulatory neurones of this complex of ganglia comprise mediator serotonin, which modulates synaptic input of command neurones. These cells are a necessary, because if their function is impaired by a selective neurotoxin 5,7-DHT (5,7-dihydroxytriptamine), both the sensitization of the withdrawal reaction and associative aversive conditioning are missing [17]. It was shown, that food-aversion learning results in significant increase of synaptic response of withdrawal command neurons to a particular type of food presentation, thus evoking the spike discharge and corresponding withdrawal [17]. Besides this, during formation of avoidance reflexes, including food aversion, an excitability and the amount of bound calcium changes in command neurons [27], [28], [29], [30]. Previously we found that in the case of food avoidance learning of adult *Helix*, following 15 minutes training, there is a significant activation of MAPK/ERK kinase and histone H3 acetylation in command neurons of the left, but not right parietal ganglion [21], [23]. The observed changes in histone H3 acetylation level in identified command neurons of parietal ganglia were less pronounced compared to our current study (where we investigated total subesaphoegal complex). This can be explained by the longer training interval in the current study and by the fact that this complex might contain other neurons, involved in avoidance learning, that potentially underwent drastic changes in acetylation. In total there are 9 command neurons located in subesophageal ganglia important for avoidance behavior (4 in parietal ganglia, 4 in pleural ganglia and 1 in visceral ganglia), there are also serotoninergic neurons essential for avoidance learning located in the pedal ganglia. A significant proportion of the neurons in this ganglion are not identified and their functions are not known.

MAPK/ERK cascade plays an important role in the induction of histone H3 acetylation in *Helix*. Earlier, we found a significant activation of MAPK/ERK in the subesophageal complex of ganglia of the adult snails after formation of food aversion learning, which was abolished by a pretreatment with MEK (ERK upstream kinase) inhibitor PD98059 [13]. Moreover, the PD98059 pretreatment prevented learning dependent histone H3 acetylation (Fig. 2A) and impaired food avoidance learning [13]. Recently, the involvement of MAPK/ERK in regulation of histone acetylation was observed in a number of studies carried out on vertebrates [6], [7], [8], [26]. It is suggested that the MAPK/ERK-dependent acetylation of histones can be mediated by the CREB-binding protein (CBP), a well-known transcription activator, which possesses endogenous histone acetyltransferase activity [7], [14]. In addition, the important role of MAPK/ERK-dependent histone H3 acetylation during formation of food aversion reflex was supported by our experiments performed in juvenile snails. The juvenile animals, possessing immature mechanisms of long-term plasticity of the avoidance behavior, in contrast to the adults, did not show histone H3 acetylation during learning. This correlates with our previous ontogenetic study, which demonstrated the lack of MAPK/ERK activation, as well as the change in the spectrum of MAPK/ERK induced transcription factors (TFs) binding DNA regulatory elements SRE and AP-1, in the juvenile animals [13], [20]. Therefore, dysfunction of MAPK/ERK activation during training may result not only in the insufficiency of phosphorylation of the downstream transcription factors, but in the deficiency of H3 histone acetylation in juvenile snails.

We suggest that the sensory stimulation does not have a significant effect on H3 histone acetylation since both adult and juvenile snails, which possess mature sensory systems [12], were treated with similar stimulations (both conditioned and unconditioned stimuli), but only in adults did we observe increased H3 histone acetylation after training. Moreover, inhibition of MAPK/ERK by PD98059 impaired food avoidance learning in adult *Helix* [13]. As described above, formation of long-term sensitization and conditioned avoidance reflexes during ontogenesis is associated with the course of development of serotoninergic system [12], [18], [19]. Currently, it is not clear whether pre- or postsynaptic link of signal transduction is responsible for dysfunction of long-term memory formation in the juveniles. Serotonin is known to appear very early in ontogenesis and participate in processes of differentiation of nervous system both in vertebrates and invertebrates, but full development of serotoninergic terminals occurs only in postnatal ontogenesis [31]. On the other hand, there are several types of serotonin receptors which play different roles in functioning of CNS and coupled with different regulatory cascades and different types of receptors appear asynchronously in ontogenesis [31], [32], [33]. Mollusks are also shown to have several types of serotonin receptors [18], but we did not find ontogenetic studies addressing this problem. We suppose that the absence of some types of serotonin receptors in juvenile snails might determine the deficiency of long-term plasticity of avoidance behavior in juvenile animals. Our observations are in agreement with the data of Stark and Carew (1999), which suggested that immaturity of facilitation mechanisms in juvenile Aplysia is not due to serotonin levels but is determined by the immaturity of its receptor chain [19]. Earlier we have shown that the neurotoxin 5,7-DHT (5,7-dihydroxytryptamine), which induces dysfunction of serotoninergic terminals and reduces conditioned food aversion learning, abolishes MAPK/ERK activation [13], [22].

Thus serotonin-dependent dysfunction of the MAPK/ERK signaling and disturbance of its downstream targets (TFs and histones) might result in the impaired expression of genes necessary for long-term plasticity and determine the deficiency of avoidance behavior plasticity in juvenile animals.

Moreover, we demonstrated that stimulation of acetylation processes in juvenile snails might promote long-term memory formation. Thus, the injection of HDAC inhibitor sodium butyrate in *Helix* significantly increases the number of avoidance responses two days after learning. Our findings are consistent with previous studies carried out on vertebrates with mental retardation, which have shown capability to improve memory through induction of the acetylation processes by injection of HDAC inhibitor [14], [16], [34]. However, most relevant to our investigation, is a study by Fischer et al. [16], which demonstrated that HDAC inhibitor sodium butyrate NaB restored spatial memory in mice even after neuronal loss, characterized by memory dysfunction. At the same time as the sprouting of dendrites an increased number of synapses are induced. Moreover, in aging mice, having significant memory impairment due to neurodegeneration, HDAC inhibitors could restore the ability to recall after in vivo injection. The mechanisms underlying the effects of HDAC inhibitors observed in both our and the Fischer et al. studies [16] are still unclear and need further investigation.

It would seem that the influence of HDAC inhibitors in long-term memory formation may be promoted through the histone-dependent chromatin remodeling, as well as through the activation of unidentified TFs. Some TFs are known to be activated through acetylation [35]. In addition, training procedures were long enough in duration to affect the MAPK/ERK signaling and downstream TFs activation through a positive feedback loop. The lack of serotonin dependent MAPK/ERK induction in juvenile animals may be compensated for through histone acetylation-dependent activation of growth factors expression [36]. These factors activate MAPK/ERK through the tyrosine-kinase receptors. These results on both MAPK/ERK dependent TFs activation and histone acetylation, which consequently lead to the activation of genes expression involved in the long-term memory formation.

We should not exclude the possibility that the activation of several genes requires the involvement of different signaling pathways in juvenile animals. The increase of acetylation can influence their induction as well.

### Conclusions

Animals’ capacities for different forms of learning do not mature simultaneously during ontogenesis but the molecular mechanisms of behind the delayed development of specific types of memory are not fully understood. We found that significantly increased MAPK/ERK -dependent histone H3 acetylation occurs in the CNS of adult animals after food aversion learning. In contrast to adult snails, juveniles possess immature plasticity mechanisms of the avoidance reflex and the histone H3 acetylation is not activated after learning. At the same time we show here that stimulation of acetylation by means of NaB in juvenile snails promoted long-term memory formation.

Thus our results suggest that the ERK-dependent histone H3 acetylation plays an essential role in food aversion learning in adult *Helix*. Dysfunction of ERK-dependent histone H3 acetylation through a lack of the expression of certain genes might determine the deficiency of long-term plasticity of avoidance behavior in juvenile animals. Such immaturity of long-term memory mechanisms can be compensated for by increasing histone acetyltransferase activity and subsequently by the induction of epigenetic changes. Our data supports and extends the idea that the molecular machinery, involved in long-term memory formation, including epigenetic tagging, is an evolutionary conserved phenomenon and can be modulated by chemical molecules.

## Methods

### Animals and Behavioral Studies

Experiments were carried out on adult (20–25 g) and juvenile (2–3 months old, 0.5–0.9 g) snails *Helix lucorum*, and did not require ethical approval. For the experiments were used adult snails from the Crimea population, which had been in the active phase not less than 4 weeks and juvenile snails which were raised in laboratory conditions until needed. All animals were kept in terrariums.

Conditioned food aversion reflex was used as a model of learning (conditioned stimulus - a piece of carrot, unconditioned stimulus - electric shock). A similar procedure was used and described in Balaban [17]. A piece of carrot (conditioned stimulus) was placed at a distance of 1 cm from the head of a snail freely moving on a metal plate (serving as one of stimulating electrodes). When a snail began to eat the carrot, another stimulating electrode was manually placed to the snail’s head, and unconditioned stimulus (DC, 1–5 mA, 0.5 s) was applied. If a snail avoided the carrot for 2 min, a piece of carrot was placed close to the snail’s mouth, and the unconditioned stimulus was applied. All trained snails received equal stimuli. The training procedure consisted of 8 pairs of stimuli applied at 15–20 min interval (4 trials per a session, 1 session per day). Animals were deprived of food for 3 days before the training. Naïve animals were used as a control group. 1 hour after training the snail’s CNS (suboesophageal complex of ganglia) was isolated. The subesophageal complex of ganglia contains neurons which participate in organization of avoidance behavior (including sensory, motor and command neurons). It was shown that about 90% of neurons in these ganglia respond to a nociceptive stimulation [17]. Prior to the isolation of the CNS, animals were anaesthetized with ice-cold saline supplemented by the injection of isotonic solution of MgCl_2_.

### Drugs and Injection Procedure

The MEK1 inhibitor PD98059 (Cell Signaling) was freshly dissolved in dimethyl sulfoxide (DMSO) at a concentration of 20 mM. Then 6 µl of PD98059 or vehicle were injected into the *cephalopedal sinus* 30 min before conditioning. The total volume of adult *Helix* hemolymph was estimated at 3 ml resulting in an approximate 500-fold dilution of the drug in hemolymph. Thus, final concentration of PD98059 in hemolymph was around 40 µM. In previous studies we showed, that PD98059 injected at this concentration impaired food avoidance learning in adult *Helix* [13].

To induce acetylation, the animals were treated with deacetylase inhibitor sodium butyrate (Sigma). Sodium butyrate (NaB) freshly dissolved in saline (80 mM NaCl; 4 mM KCl; 7 mM CaCl_2_; 5 mM MgCl_2_·6H_2_O; 5 mM Tris) was injected at a dose of 1,2 mg/g of animal weight (0.01 ml). Injections were made into the *cephalopedal sinus* 1 h prior conditioning. Control animals were injected with saline only. Injections were given on both training days. Animals were tested 48 hours after the learning procedure.

### Extraction of Histones and Immunoblotting

To identify histone acetylation status, CNS were homogenized in extraction buffer: 10 mM Tris-HCl pH7.5, 1 mM EDTA, 2.5 mM sodium pyrophosphate, 1 mM β-glycerophosphate, 0.2 mM PMSF, 1% protease inhibitor cocktail (Sigma), 0.1 mM Na_3_VO_4_, and 1% Igepal CA-630. Histones were extracted according to Levenson [6]. All procedures were performed on ice. Tissue homogenates were centrifuged at 7,700×*g* for 5 min (4°C). The pellet was resuspended in 1 ml of 0.4 N H_2_SO_4_ (and was incubated for 30 minutes for the histone extraction) and then centrifuged at 14,000×*g* for 10 min (4°C). The supernatant was transferred to a new tube, and proteins were precipitated with the addition of 250 µl of 100% trichloroacetic acid containing 4 mg/ml deoxycholic acid (Na^+^ salt, Helicon) for 30 min and then centrifuged at 14,000×*g* for 30 min (4°C). The supernatant was discarded and the protein pellet was washed with 1 ml of acidified acetone (0.1% HCl) followed by 1 ml of acetone for 5 min each. Protein precipitates were collected by centrifugation (14,000×*g*, 5 min, 4°C). This procedure was done 3 times. The resulting purified proteins were resuspended in 10 mM Tris (pH 8.0) and stored at −80°C.

Protein concentration was measured by Bradford assay. Samples (1 µg) were boiled with loading buffer and equal amount of protein was loaded into the 14% SDS-PAGE. Protein markers were from Fermentas (Lithiania). Separated proteins were transferred to the nitrocellulose membrane (Schleicher and Schuel). Ponceau S staining was used to check transfer quality. Membranes were incubated in Tris-buffered saline (pH 7.6) with 0.1% Tween 20 (TBS-T) containing 5% non-fat dry milk for 1 h at 4°C to block nonspecific binding. Subsequently blots were incubated with primary Acetylated-H3-Histone antibodies (4°C overnight) and with secondary antibodies conjugated with HRP (horseradish peroxidase) for 1 h. Immunolabeling was detected by enhanced chemiluminescence using ECL system (standard protocol and components from «Amersham pharmacia biotech»). Blots were then stripped (glycine-HCl, pH 2.8, two times for 20 min each at 55°C), saturated 1 hour in 5% nonfat dry milk and incubated with antibodies against total form of histone H3. After exposure of the membranes, films were scanned and the amount of protein was quantified using the Gel Pro Anal computer program.

### Antibodies

The amount of acetylated histone H3 was calibrated to the amount of total histone H3, the level of which remains stable in learning. To visualize H3-histone acetylation polyclonal antibody against Acetylated-H3-histone (Cell Signaling) was used. Polyclonal antibody against total histone H3 (Cell Signaling) was used for analysis of H3 content. Antibodies against Acetylated-H3-histone and total histone H3 were diluted 1∶1000 and secondary antibodies (Amersham) were diluted 1∶1500–1∶2500.

#### Data analysis

Statistical analysis was carried out with ANOVA followed by Scheffe’s, Fischer’s and Tukey’s test for post-hoc comparison. Significance of results was accepted at *p*≤0.05. The results are presented as mean ± SEM. Cross-tabulation tables with 2 sided Fisher’s exact test were used for comparison of probabilities of behavior.
